# Supplementary material for: Chiral DNA sequences as commutable controls for clinical genomics
Source: Nat Commun. 2019 Mar 22;10:1342. doi: 10.1038/s41467-019-09272-0 (PMC6430799; doi:10.1038/s41467-019-09272-0)
Supplement: Supplementary file 1 — Supplementary Information [file 41467_2019_9272_MOESM1_ESM.pdf]

SUPPLEMENTARY INFORMATION

Deveson et al:

Chiral DNA sequences (sequins) as commutable controls for clinical genomics

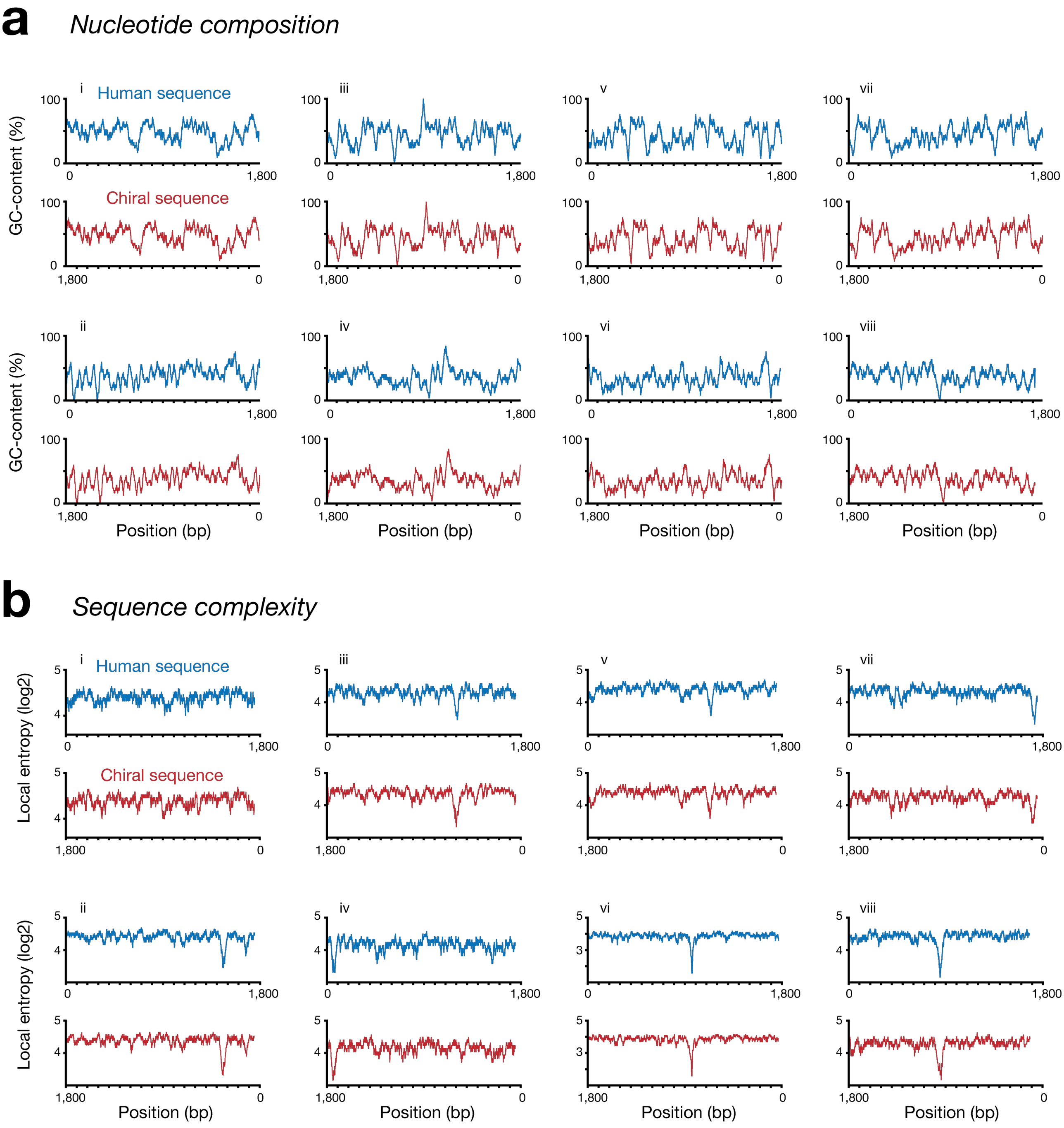

Supplementary Fig. 1. Matched sequence properties of chiral DNA sequence pairs. (**a**) Local GC-content (%) and (**b**) sequence complexity of eight human (*fwd;* blue) and chiral (*rev*; red) DNA sequence pairs. Local GC-content was calculated using a 25 bp sliding window. Sequence complexity is represented by topological entropy, calculated as per Orlov and Potapov (2004)[1].

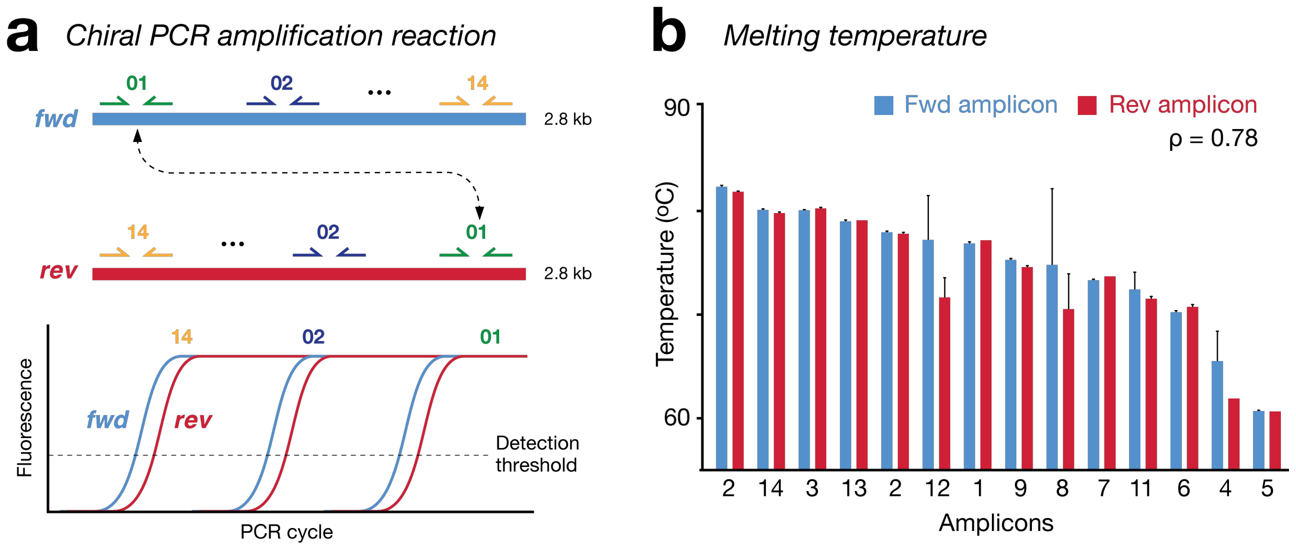

Supplementary Fig. 2. Matched performance of chiral DNA sequence pairs during PCR amplification. (**a**) Schematic showing primer pairs targeting 14 random, non-overlapping intervals within a 2.8 kb human (*fwd*) DNA template, and mirrored primers targeted the corresponding sequences in its corresponding chiral (*rev*) DNA template, thereby creating 14 chiral pairs of PCR amplicons (**Supplementary Table 1**). Human and chiral DNA templates were combined at equal concentration and real-time PCR was performed using each primer pair. Given they are amplified from a common template, the order of detection among *fwd* amplicons, and among *rev* amplicons, indicates the relative amplification efficiencies within each orientation category. (**b**) Box plot presents melting temperatures recorded for human (*fwd;* blue) and chiral (*rev;* red) amplicon pairs during real-time PCR analysis.

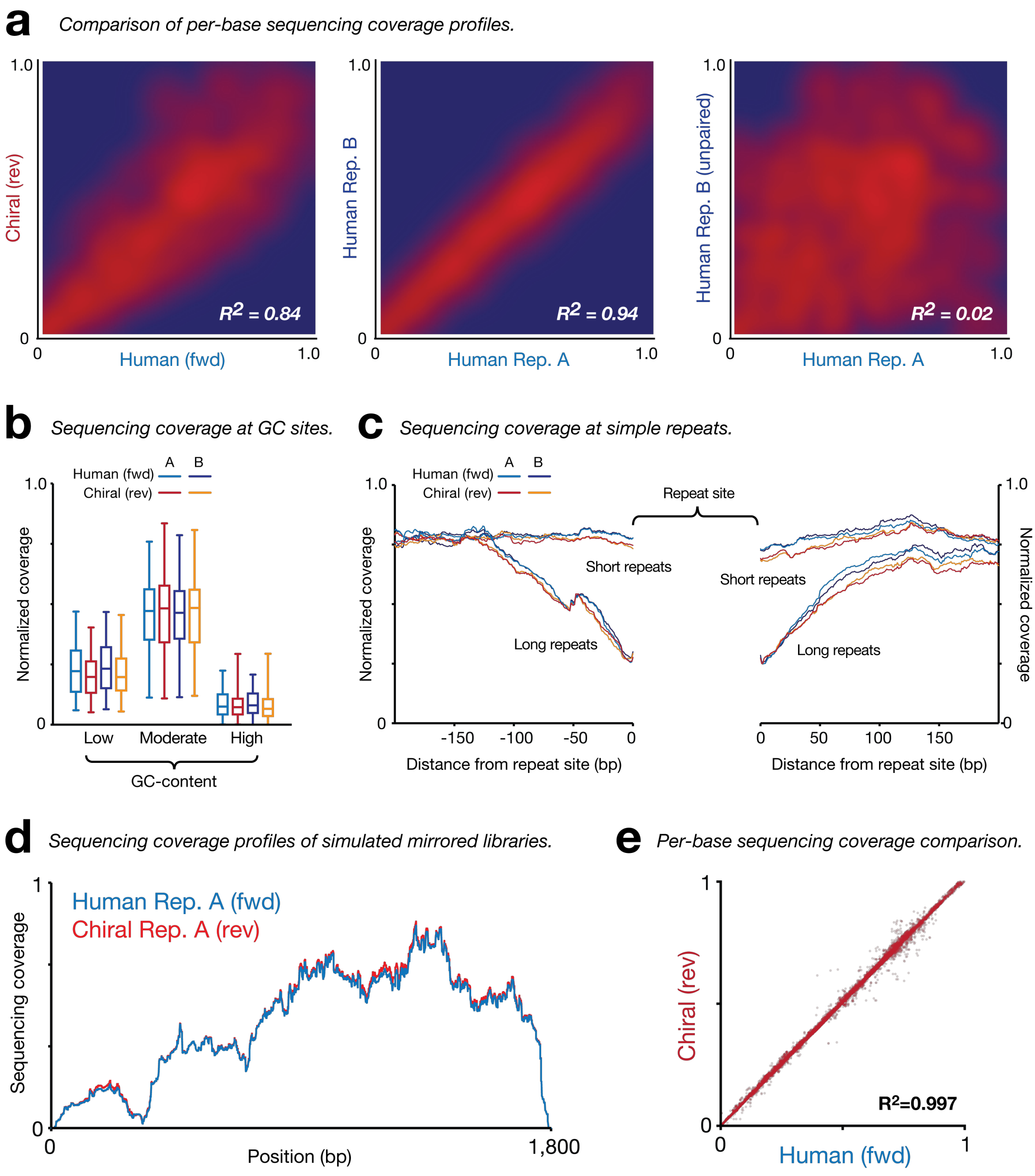

Supplementary Fig. 3. Matched sequencing coverage profiles of chiral DNA sequence pairs. (**a**) Density scatter plots show the concordance of per-base sequencing coverage profiles for paired human and chiral (*fwd/rev*) synthetic DNA sequences (left), analyzed by next generation sequencing (NGS). For comparison, the concordance of identical human (*fwd/fwd*) sequences (center) and un-paired sequences (right) between replicate NGS experiments is also shown. (**b**) Normalized coverage within local regions of low (<20%), moderate and high (>70%) GC-content in human and chiral sequences. (**c**) Normalized sequencing coverage aggregated with respect to simple repeat sites in human and chiral sequences. Repeats are divided into 'short' (5-9 units for mono- and dinucleotides, 3-6 units for tri- and quadnucleotide repeats) and 'long' (≥10 units for mono- and dinucleotides, ≥7 units for tri- and quadnucleotide repeats). (**d**) Normalized sequencing coverage within a single synthetic chiral DNA sequence pairs (1.8 kb), where each human read is matched by a simulated chiral equivalent. (**e**) Density scatter plot shows the concordance of per-base coverage profiles between simulated mirrored sequencing libraries.

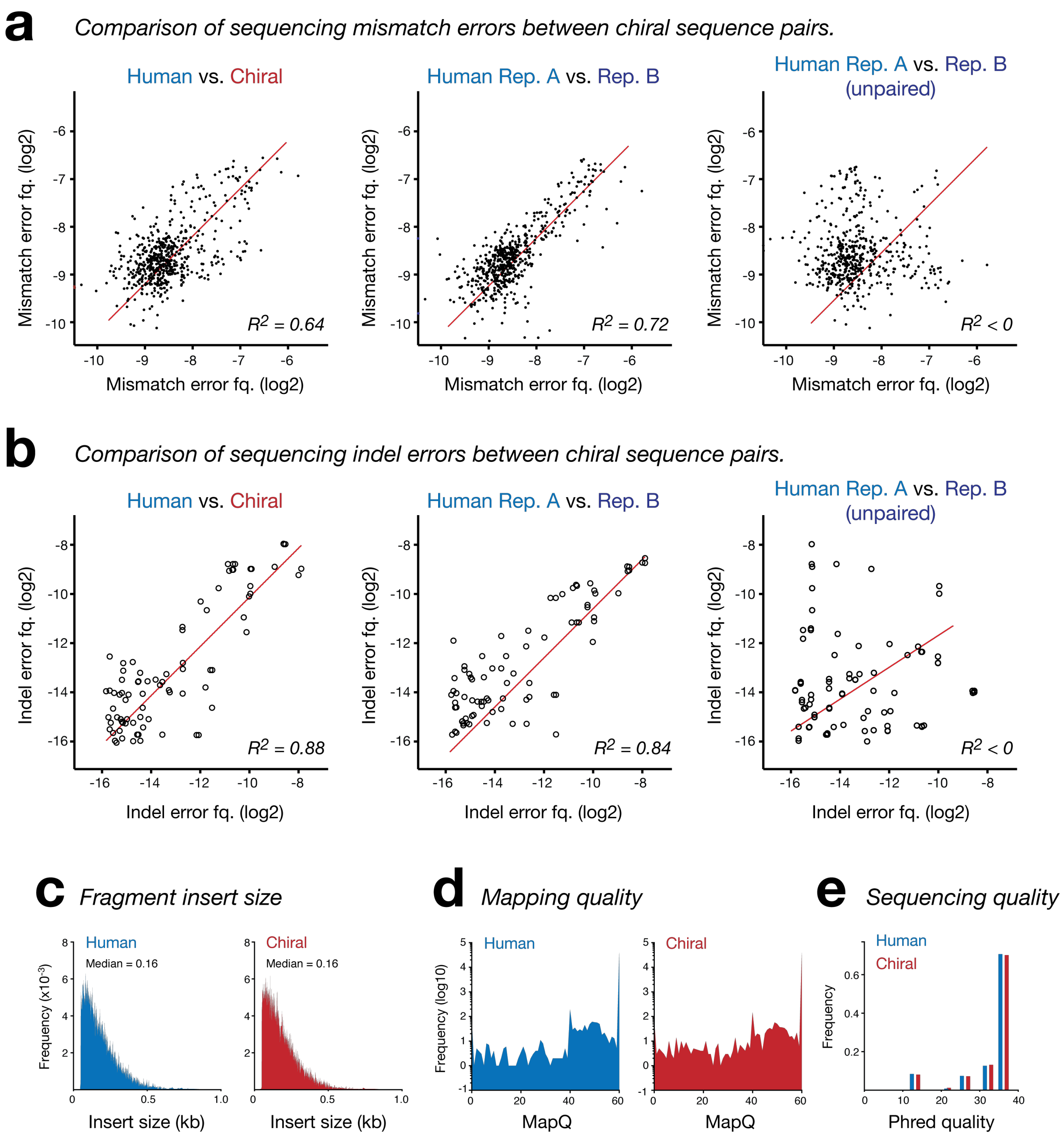

Supplementary Fig. 4. Matched sequencing error profiles of chiral DNA sequence pairs. (**a**,**b**) Scatter plots show concordance of sequencing error frequency profiles between human and chiral (*fwd/rev*) DNA sequence pairs (left). For comparison, the concordance of identical human (*fwd/fwd*) DNA sequences (center) and un-paired sequences (right) between replicate experiments is also shown. Mismatch error profiles (**a**) were calculated using a 25nt sliding window and indel errors (**b**) were calculated using 50nt sliding window. (**c-e**) Population distributions for fragment insert size (**c**), mapping quality (**d**) and sequencing quality (**e**) obtained for human/chiral synthetic DNA sequence pairs.

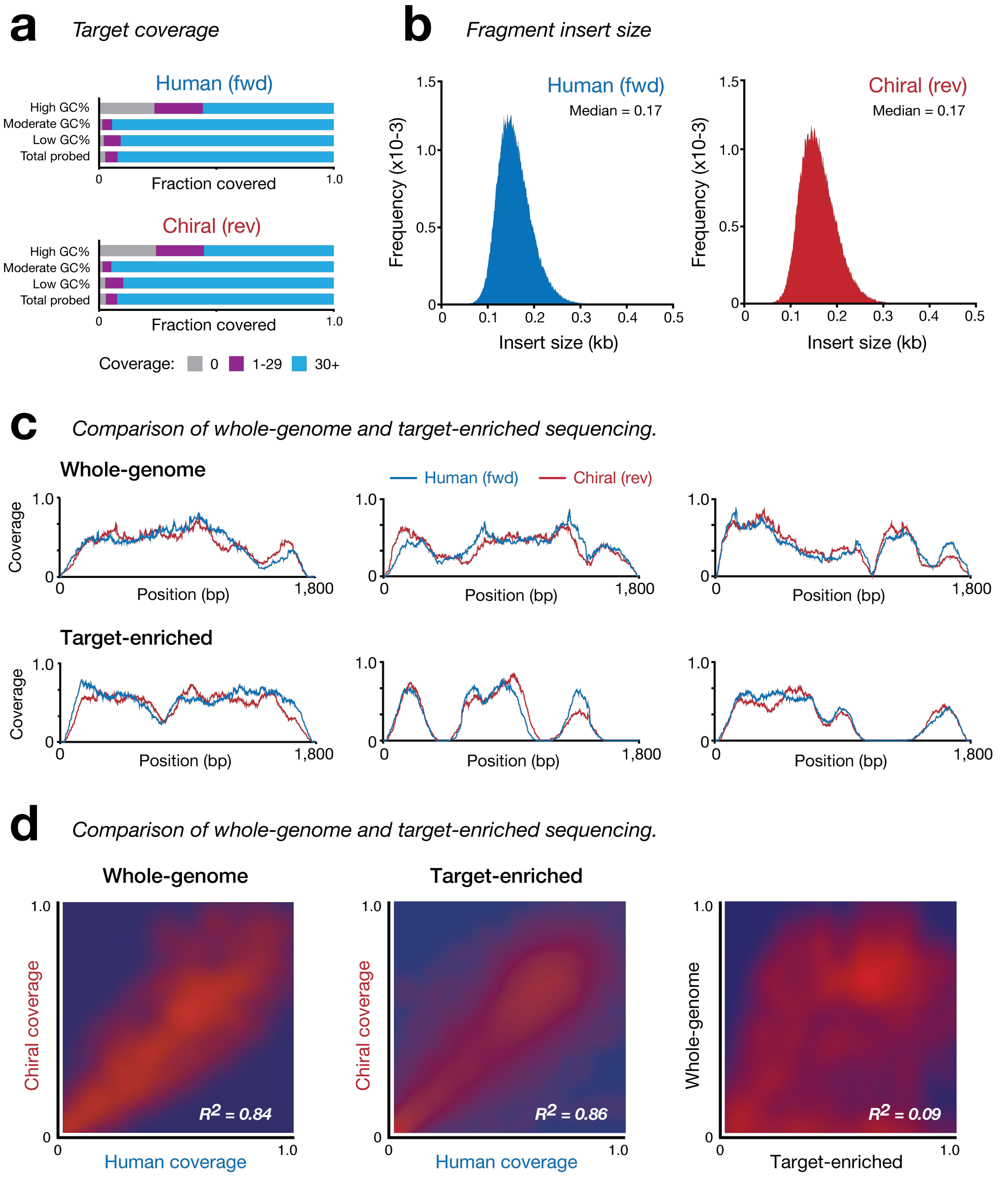

Supplementary Fig. 5. Divergent technical biases of whole-genome and target-enriched sequencing. (**a**) Fraction of targeted regions with 0 (grey), 1-29 (purple) or ≥30 (blue) alignment fold-coverage for human genome regions (upper) and corresponding chiral DNA sequences (lower). (**b**) Insert fragment size distribution for human (*fwd*; blue) and chiral (*rev*; red) sequencing reads during target-enriched NGS. (**c**) Normalized coverage across three synthetic human and chiral (*fwd/rev*) sequence pairs, analyzed by whole-genome (upper) and target-enriched (lower) sequencing. (**d**) Density scatter plots show the concordance of per-base coverage profiles for paired human and chiral DNA sequences analyzed by whole-genome (left) and target-enriched (center) sequencing. Comparison of matched sequences between whole-genome and target-enriched sequencing also shown (right). The low concordance of the cross-method comparison illustrates the scale of technical variation between methods.

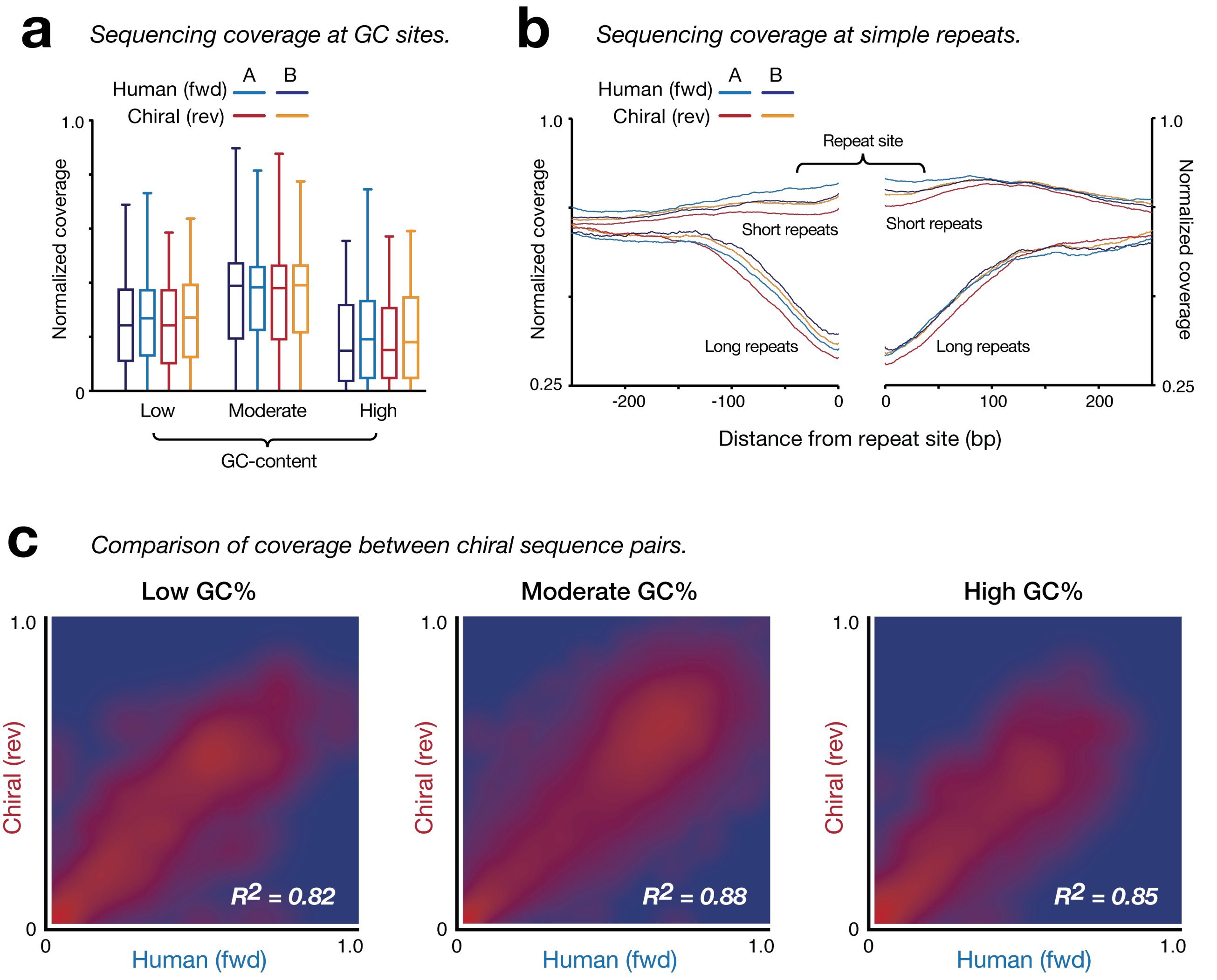

Supplementary Fig. 6. Impact of challenging sequence features on target-enriched NGS. (**a**) Human/chiral coverage concordance within local regions of low (<20%; left), moderate (center) and high (>70%; right) GC-content. (**b**) Relative coverage aggregated with respect to simple repeat sites in human and corresponding chiral sequences analyzed by target-enriched NGS. Repeats are divided into 'short' (5-9 units for mono- and dinucleotides, 3-6 units for tri- and quadnucleotide repeats) and 'long' (≥10 units for mono- and dinucleotides, ≥7 units for tri- and quadnucleotide repeats). (**c**) Density scatter plots show the concordance of per-base coverage profiles between human (*fwd*) and chiral (*rev*) DNA sequences with low (<20%), moderate, and high (>70%) GC-content.

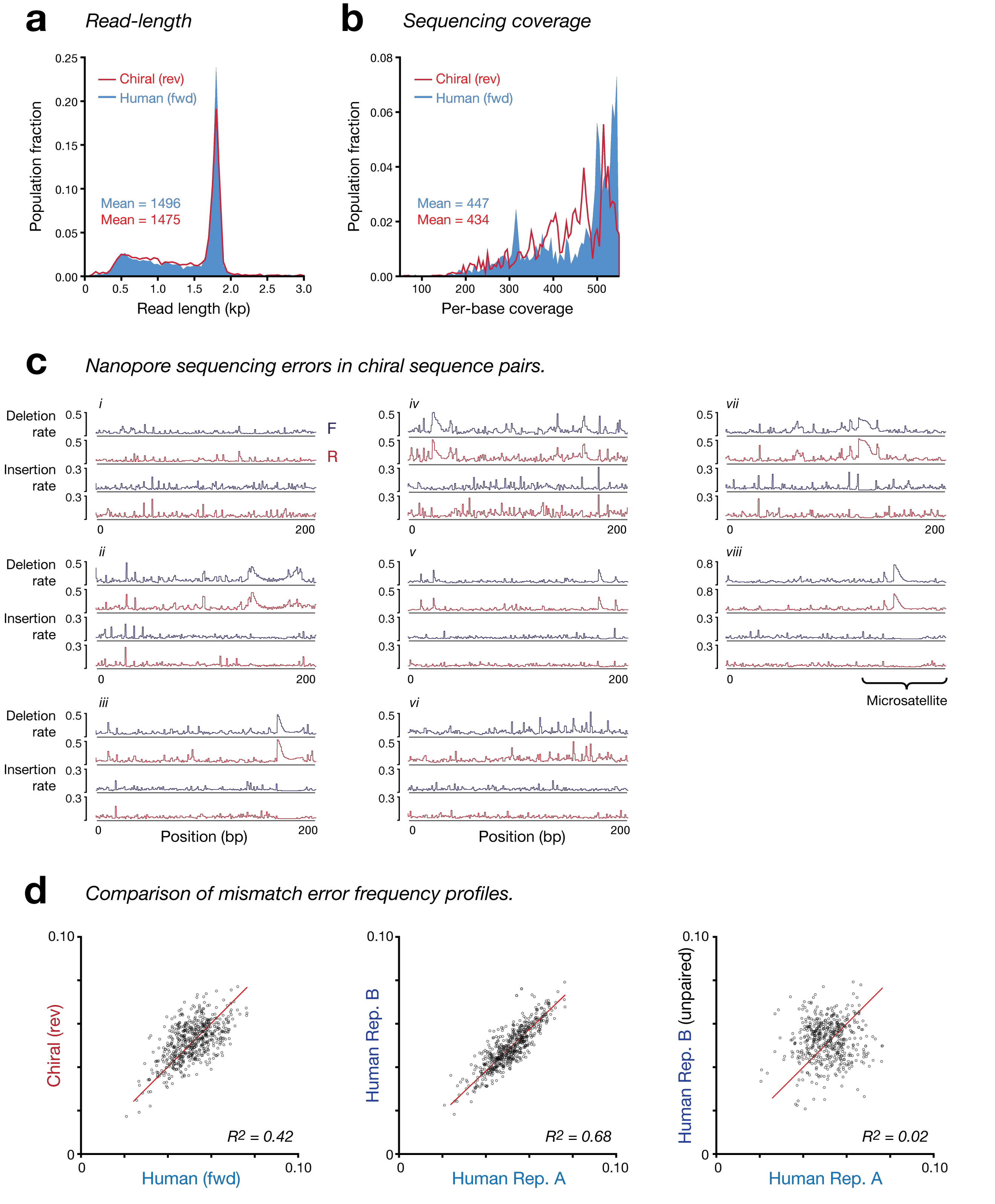

Supplementary Fig. 7. Matched performance of chiral DNA sequence pairs during nanopore sequencing. (**a**,**b**) Population distributions for read length (**a**) and per-base coverage (**b**) obtained during nanopore sequencing analysis of synthetic human (*fwd*) and chiral (*rev*) DNA sequences pairs. In each case, read-pairs deriving from human (blue) and chiral (red) sequences are shown separately for comparison. (**c**) Per-base insertion and deletion error frequencies during nanopore sequencing across a 200nt window selected from each *fwd/rev* chiral DNA sequence pair (n = 8). (**d**) Scatter plots show concordance of nucleotide mismatch error frequency profiles between *fwd/rev* chiral pairs (center). For comparison, the concordance of identical *fwd/fwd* sequences (left) and un-paired *fwd/fwd* sequences (right) sequenced on separate flow cells (technical replicates) also shown.

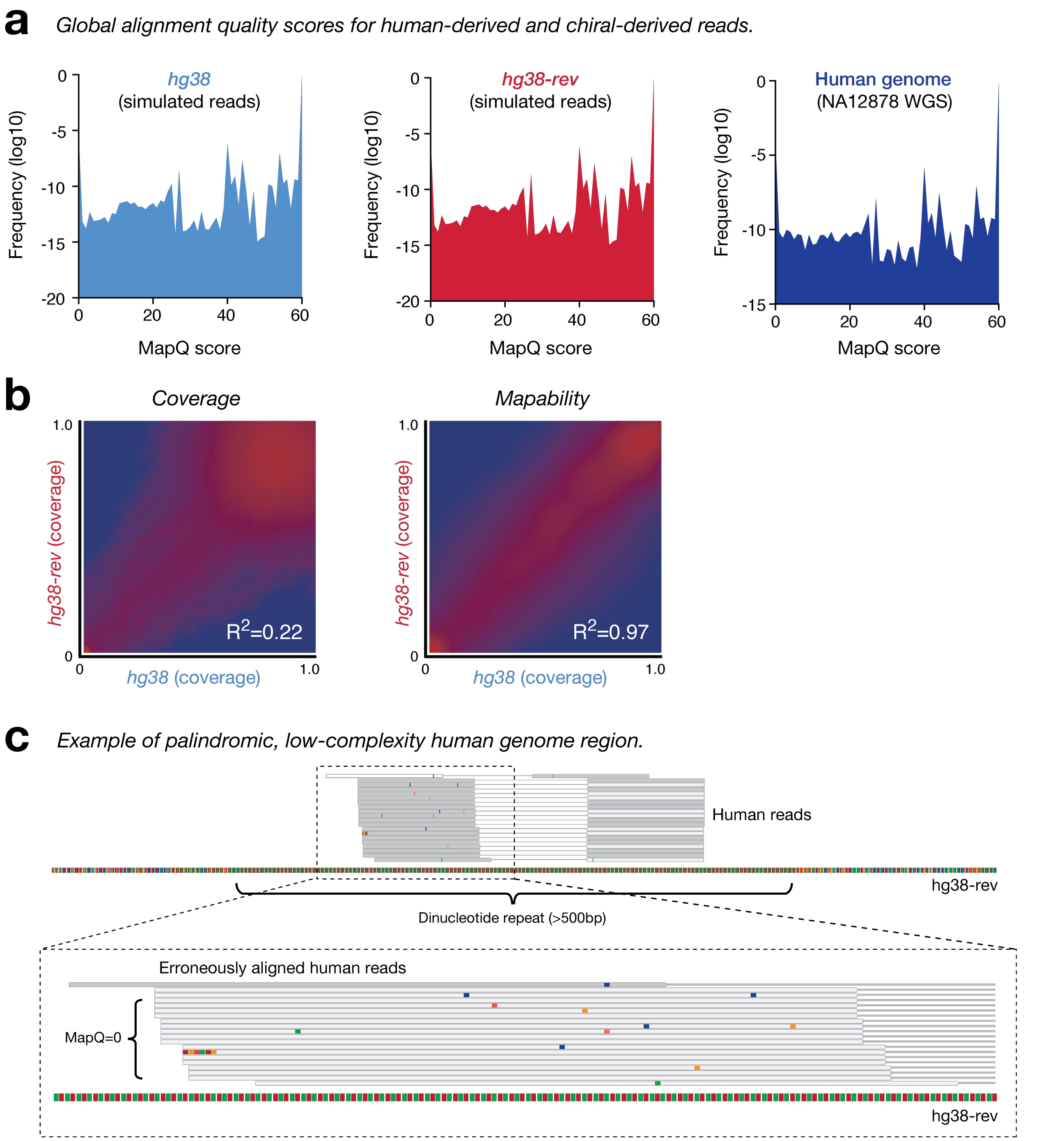

Supplementary Fig. 8. Matched alignability of chiral DNA sequence pairs. (**a**) Mapping quality distribution for simulated NGS libraries from human (*hg38;* blue), chiral (*hg38-rev;* red) and experimental human whole-genome NGS library (NA12878; navy), aligned to corresponding reference genome sequences. (**b**) Density scatter plots show the concordance of per-base coverage, and corrected sequence mapability profiles for paired regions within *hg38* and *hg38-rev*. (**c**) Example of a long dinucleotide repeat that forms palindromic sequence and violates chirality. Simulated reads from *hg38-rev* that erroneously aligned to *hg38* are shown.

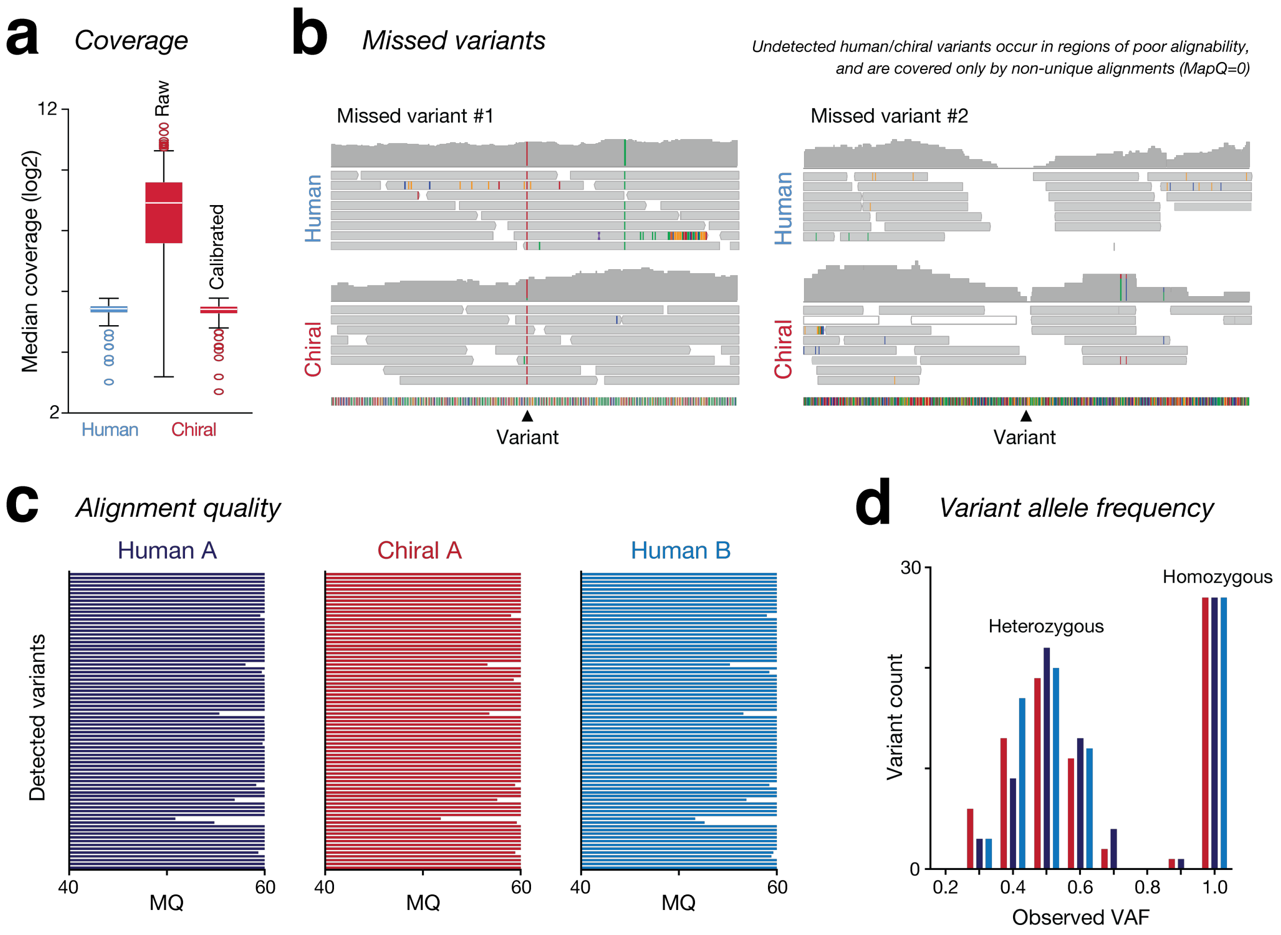

Supplementary Fig. 9. Matched properties of human and chiral genetic variants. (**a**) Coverage within genome regions represented by synthetic chiral DNA standards (n = 87), for human (navy) and chiral (*rev;* red) alignments, as analyzed by whole-genome NGS. Chiral (*rev*) DNA is sequenced in excess (raw), then down-sampled to match human genome coverage (calibrated). (**b**) Screen shot examples show the two human variants, and their chiral counterparts, that eluded detection during whole-genome NGS analysis. Both variants are in regions of low alignability, covered only by non-unique (MapQ = 0) alignments, meaning they could not be called. (**c**) Box plots show the concordance of alignment quality (MQ) scores between human variants (navy, blue) and synthetic chiral equivalents (red; matched order). (**d**) Histogram shows observed variant allele frequencies (VAFs) for human and chiral variants.

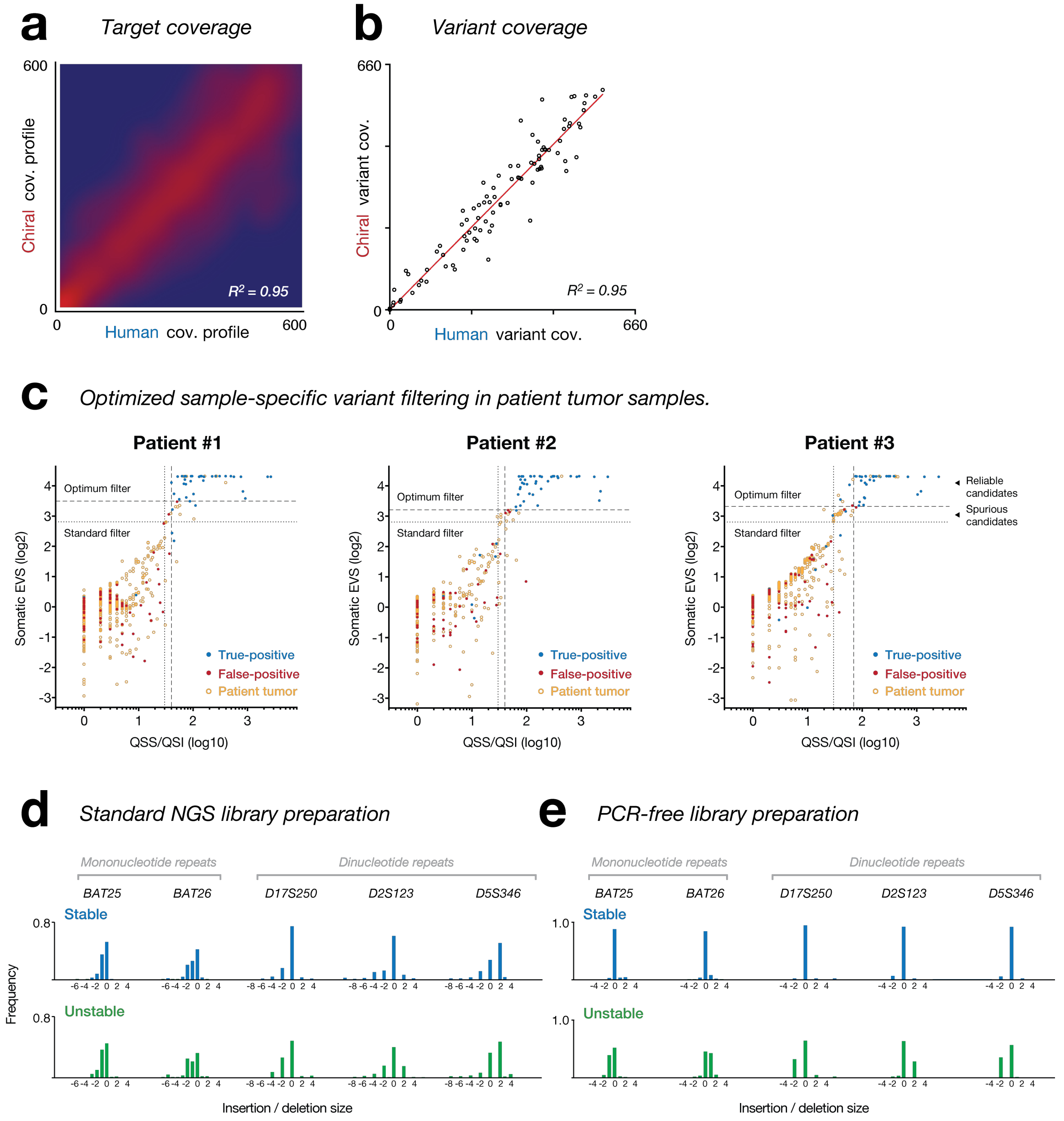

Supplementary Fig. 10. Use of chiral DNA sequins for diagnosis of cancer mutations and microsatellite instability. (**a**) Density scatter plot shows concordance of per-base sequencing coverage profiles between cancer genes and corresponding chiral DNA sequins analyzed by target-enriched NGS. (**b**) Scatter plot shows the concordance of alignment coverage at sites of cancer variants (n = 94) between cancer DNA reference sample and accompanying sequins. (**c**) Scatter-plots show confidence scores (QSS/I and SomaticEVS) for somatic variant candidates for each of three patient tumor samples and internal sequin controls. Sample-specific, optimized quality thresholds (dashed lines) exclude all false-positives (red), while retaining maximum true-positives (blue) and human variant candidates (orange). Conventional best-practice thresholds (dotted lines) are shown for comparison. (**d,e**) Histograms show the frequency of insertions and deletions in alignments, relative to the reference repeat length, at each microsatellite site for stable (upper) and unstable (lower) chiral DNA sequins mixtures. Notably, no clear distinction in insertion/deletion frequency can be observed at microsatellite sites using standard library preparation method (**d**), whilst a distinction in insertion/deletion frequency is observed using PCR-free library preparation method (**e**).

Human *(fwd)* template:

5′ –
GCCTCAAACTCTCCCCTGATGGCAGCAGAGGTGAAAAGAGAAGTTTCTCTTGGTGCCCCTTTTAAAAGCCATGCACTGTGCTGAATCATGATGTGAAAGAGTCTAACCACATGGTTCTCAACCCTCGCTGCACATTAAAATCACT
TGGGGGCTTAAAGGAAAATACTGCCAGGGACCCAACCTCAGAGATTCTCATTCAATTGCAGGTGAGGCCTGGGGATCAGTGAGGGACAGGAACTTAGACTGAGCAGCTGCTTCCTATGATGGGCCCTATGCCTTGAGCCCACCCT
GTCTGCCTCCAAAGTCCATGCCCTCCCCTGTCACCACTCTGCCCTCCAACCTGAGTCTCCCTCTGGAGTGTTCCTCCCCATCCACTGTGAGTATCAAACATGAAAGGCCCTGTTCAGTCGGGTGACTATCCACTCCTGCCACATC
ACCCACTTCCTCCCTGTGGACCAGCCCAGTCCTGGACAGTCTTGGACACCCCCAGGACATTCCTGCCATTGTCTTGAGCCTGCATGACCCCTCTCCCACCTTCTACCAAAGGGTTCTTCTTTATCATCACATACCTTTTACAGTG
TCAATGTTATTCAACAAGCACATTTTCATTTTTATTTGTTTTAAACTTTTCACTCTGAAAGAAAATTTAAGACTCACTTAAAAATTTTAAAATAGTTCAAACCAGATGTGGTGGTGCACGCCTATCATCCCAGCTACGTGGGTA
AGATTCTCAGGTGGGAGGATTACCTGAGCCCAGGGAGGTCGAGGCTGCAGCGAGCCAGGATTGTGCCACCACACTCCAGCCTGGGTGACAAAGGGAGACCCTGTCTCCAAAAAAAAAAAAAATTGTTCAGAGAATTCTTTTTTTT
TTTTTTTGGTAATGAAAACCACAATTTCGTCATTGGGTGCCACCCGTTTTTGCTTAGATTCACACTAGTTCCTTAGTGCTAATTAGCAGATACTGAAATGATCTTGACAGACTGCGCAAAATATGGAACGCTTCACAAATTTGCG
TGTCATCCTTGCGCAGGGGCCATGCTAATCTTCTTGTTCAGAGAATTCTTGTGTACCGATCAGCCAGCTTCCCTCAGTGATAACATGTTATATTACAATAGTGCAATTGTCAAAAACAGAAAAACTGACATGGTAATACTATTAA
CTAAACTACAGACCTCACAGAGATTTCACAGGTTTTTTTTTTTTTTTTTTTTGAACATAAACTCAAGATTTTATTGTCTTCATAATAAAAGATGACACTTAGAACTGGATCACTTGGCCCTTTCTCTTCTTATCTCCTCCCAGTT
CAAAATGCTTGCATCTTTTAATAGCCAGCATTCTCTTAGATCTGCAGTTGGGCTCAACGCACTCAAGCCTTAGCACAATCTTCTTTGTAGTTTTAGCCTTTTTCCGGAAAATCGGCTTAGTTTGCCCACCATAGCCACTCTGCTT
CCTGTCATAACGCCGCTTTCCCTGGGCGTACAGAGAATCCTTGCCCTTCTTGTACTGTGTCACTTTATGGGGTTGGTGCTTGCCACACTTCTTACAGAAAGTCCGGCGGGTTTTAGGGACGTTAACCATGCTTGCGTGAGCGCTA
TCGGCGCGGAAAGAAGCCAGGTTTTATATATACTATGTATATATGTACAGTTCTATGAGATTTCATCACATGCATACATTCATGTGTGCCCCACCACAATCAGGACACAGGACAGTTCCACCACTTCAAAGAAATGTACCCTCT
TCTAAGTCTTCCGCTGATCTGTTCTCCATTACTATAATTTTGTCATTTCAAGAATTTTTTATCAATGAAATAATATACTATACAACCTTTTGAGACTAGCTTTTTTAAAAATTCAACATAAGCATGTATTTATTTTGTGATCAGC
TACCACAATAAAGATACTCCCTTTTGGAAGACAACTCCAAAAAATTTTATCGATTACTGGCTAAGTAAATCGTAATTCAACTGTATAATGAAATCGTATGCAGCCATTAAAACTGATGAGATAAACATAAATTAAGGACATGTAT
ATTTTTTCAAATACACAGTCAGCTCTCCATATCCATGGGTTCCATGTCAGAGAACCCATGGATATGGAGGCCAACTGTAAGAAACTTGAGCATCCACTGATTTGCTATCTGTGGGGGCTCCTGGAACCAATCCCCAATGGATGC
CGAGAGAGAAAACTATATAGTTAAGTGAGAAAATACAAAACTATATATTATCCCATTTTTGTTAAAACACACATATAAATACATAGATACACAGAGATAGACAACAAGATGTTAACAGGGATTATCTCTGGGTGATAGGATTACA
AGGGATTTTTATTTTCTTGTTGCTTGTCTGCATTGTCTAAATTTACAACAGTGAATATAAGTAACTTTATGACTTTTTTAAATGCTTGAAGTAAAGCTCCAATTTTGCCATCTGTAACTCTTCCAGCCACAATCACGTTATTCTC
TCAGCTAGCAAAGTATTGTGTGTGGGTCCCCATTCTAGGACTCACATCATTCAGAGGCTCAGCATAGCTGCTTGTGGGCTAGCCGGCCCCCACCTCAGGAAGGGGCTGTACTCATGAATGGTCTGCATCCCTCTGGCCCCTTTTG
GCTCAGCCCTTGCCAGGCATAGGTACTCATCAATGGATCTGAGGCTGCAACCATACCAGGAGCTATTTGGCCAGAGCCCCAAGGCTCAAAAAGAAAGCCCCTGGAATTTCTCAGGGTATTTGGCATTCTAGAGTGAGTGACATCT
TGGTCCCCACCACCTAGGCCTTAAGAAATAAGGCCTGGGAGTAAG

Chiral (rev) template:

5′ –
GAATGAGGGTCCGGAATAAAGAATTCCGGATCCACCACCCCTGGTTCTACAGTGAGTGAGATCTTACGGTTTATGGGACTCTTTAAGGTCCCCGAAAGAAAAACTCGGAACCCCGAGACCGGTTTATCGAGGACCATACCAACGT
CGGAGTCTAGGTAACTACTCATGGATACGGACCGTTCCCGACTCGGTTTTCCCCGGTCTCCCTACGTCTGGTAAGTACTCATGTCGGGGAAGGACTCCACCCCCGGCCGATCGGGTGTTCGTCGATACGACTCGGAGACTTACTA
CACTCAGGATCTTACCCCTGGGTGTGTGTTATGAAACGATCGACTCTCTTATTGCACTAACACCGACCTTCTCAATGTCTACCGTTTTAACCTCGAAATGAAGTTCGTAAATTTTTTCAGTATTTCAATGAATATAAGTGACAAC
ATTTAAATCTGTTACGTCTGTTCGTTGTTCTTTTATTTTTAGGGAACATTAGGATAGTGGGTCTCTATTAGGGACAATTGTAGAACAACAGATAGAGACACATAGATACATAAATATACACACAAAATTGTTTTTACCCTATTAT
ATATCAAAACATAAAAGAGTGAATTGATATATCAAAAGAGAGAGCCGTAGGTAACCCCTAACCAAGGTCCTCGGGGGTGTCTATCGTTTTAGTCACCTACGAGTTCAAAGAATGTCAACCGGAGGTATAGGTACCCAAGAGACTG
TACCTTGGGTACCTATACCTCTCGACTGACACATAAACTTTTTATATGTACAGGAATTAAATACAAATAGAGTAGTCAAAATTACCGACGTATGCTAAAGTAATATGTCAACTTAATGCTAAATGAATCGGTCATTAGCTATTT
TAAAAAACCTCAACAGAAGGTTTTCCCTCATAGAAATAACACCATCGACTAGTGTTTTATTTATGTACGAATACAACTTAAAAATTTTTTCGATCAGAGTTTTCCAACATATCATATAATAAAGTAACTATTTTTTAAGAACTTT
ACTGTTTTAATATCATTACCTCTTGTCTAGTCGCCTTCTGAATCTTCTCCCATGTAAAGAAACTTCACCACCTTGACAGGACACAGGACTAACACCACCCCGTGTGTACTTACATACGTACACTACTTTAGAGTATCTTGACATG
TATATATGTATCATATATATTTTTGGACCGAAGAAAGGCGCGGCTATCGCGAGTGCGTTCGTACCAATTGCAGGGATTTTGGGCGGCCTGAAAGACATTCTTCACACCGTTCGTGGTTGGGGTATTTCACTGTGTCATGTTCTTC
CCGTTCCTAAGAGACATGCGGGTCCCTTTCGCCGCAATACTGTCCTTCGTCTCACCGATACCACCCGTTTGATTCGGCTAAAAGGCCTTTTTCCGATTTTGATGTTTCTTCTAACACGATTCCGAACTCACGCAACTCGGGTTGA
CGTCTAGATTCTCTTACGACCGATAATTTTCTACGTTCGTAAAACTTGACCCTCCTCTATTCTTCTCTTTCCCGGTTCACTAGGTCAAGATTCACAGTAGAAAATAATACTTCTGTTATTTTAGAACTCAAATACAAGTTTTTTT
TTTTTTTTTTTTTGGACACTTTAGAGACACTCCAGACATCAAATCAATTATCATAATGGTACAGTCAAAAAGACAAAAACTGTTAACGTGATAACATTATATTGTACAATAGTGACTCCCTTCGACCGACTAGCCATGTGTTCTT
AAGAGACTTGTTCTTCTAATCGTACCGGGGACGCGTTCCTACTGTGCGTTTAAACACTTCGCAAGGTATAAAACGCGTCAGACAGTTCTAGTAAAGTCATAGACGATTAATCGTGATTCCTTGATCACACTTAGATTCGTTTTTG
CCCACCGTGGGTTACTGCTTTAACACCAAAAGTAATGGTTTTTTTTTTTTTTTTCTTAAGAGACTTGTTAAAAAAAAAAAAAACCTCTGTCCCAGAGGGAAACAGTGGGTCCGACCTCACACCACCGTGTTAGGACCGAGCGACGT
CGGAGCTGGAGGGACCCGAGTCCATTAGGAGGGTGGACTCTTAGAATGGGTGCATCGACCCTACTATCCGCACGTGGTGGTGTAGACCAAACTTGATAAAATTTTAAAAATTCACTCAGAATTTAAAAGAAAGTCTCACTTTTCA
AATTTGTTTATTTTTACTTTTACACGAACAACTTATTGTAACTGTGACATTTTCCATACACTACTATTTCTTCTTGGGAAACCATCTTCCACCCTCTCCCCAGTACGTCCGAGTTCTGTTACCGTCCTTACAGGACCCCCACA
GGTTCTGACAGGTCCTGACCCGACCAGGTGTCCCTCCTTCACCCACTACACCGTCCTCACCTATCAGTGGGCTGACTTGTCCCGGAAAGTACAAACTATGAGTGTCACCTACCCCTCCTTGTGAGGTCTCCCTCTGAGTCCAACC
TCCCGTCTCACCACTGTCCCCTCCCGTACCTGAAACCTCCGTCTGTCCCACCCGAGTTCCGTATCCCGGGTAGTATCCTTCGTCGACGAGTCAGATTCAAGGACAGGGAGTGACTAGGGGTCCGGAGTGGACGTTAACTTACTCT
TAGAGACTCCAACCCAGGGACCGTCATAAAAGGAAATTCGGGGGTTCACTAAAATTACACGTCGCTCCCAACTCTTGGTACACCAATCTGAGAAAGTGTAGTACTAAGTCGTGTCACGTACCGAAAATTTTCCCCGTGGTTCTCT
TTGAAGAGAAAAGTGGAGACGACGGTAGTCCCCTCTCAAACTCCG

Primer name	Sequence (5′-3′)	Primer name	Sequence (5′-3′)
FWD_1p1	CTCTCCCCTGATGGCAGCAG	REV_1p1	CGCTCCCAACTCTTGGTACA
FWD_1p2	GCGAGGGTTGAGAACCATGT	REV_1p2	GAGAGGGGACTACCGTCGTC
FWD_2p1	GGCCTGGGGATCAGTGAGGG	REV_2p1	TCTCACCACTGTCCCCTCCC
FWD_2p2	AGAGTGGTGACAGGGGAGGG	REV_2p2	CCGGACCCCTAGTCACTCCC
FWD_3p1	TCTGGAGTGTTCCTCCCCAT	REV_3p1	AGGTCCTGACCCGACCAGGT
FWD_3p2	TCCAGGACTGGGCTGGTCCA	REV_3p2	AGACCTCACAAGGAGGGGTA
FWD_4p1	TCTCCCACCTTCTACCAAAG	REV_4p1	AAAAGAAAGTCTCACTTTTC
FWD_4p2	TTTTCTTTCAGAGTGAAAAG	REV_4p2	AGAGGGTGGAAGATGGTTTC
FWD_5p1	TTTAAAATAGTTCAAACCAG	REV_5p1	TAGGACCGAGCGACGTCGGA
FWD_5p2	ATCCTGGCTCGCTGCAGCCT	REV_5p2	AAATTTTATCAAGTTTGGTC
FWD_6p1	GACAAAGGGAGACCCTGTCT	REV_6p1	CTTAGATTCGTTTTTGCCCA
FWD_6p2	GAATCTAAGCAAAAACGGGT	REV_6p2	CTGTTTCCCTCTGGGACAGA
FWD_7p1	TCTTGTGTACCGATCAGCCA	REV_7p1	AGACACTCCAGACATCAAAT
FWD_7p2	TCTGTGAGGTCTGTAGTTTA	REV_7p2	AGAACACATGGCTAGTCGGT
FWD_8p1	GATTTTATTGTCTTCATAAT	REV_8p1	TAGATTCTCTTACGACCGAT
FWD_8p2	ATCTAAGAGAATGCTGGCTA	REV_8p2	CTAAAATAACAGAAGTATTA
FWD_9p1	TTAACCATGCTTGCGTGAGC	REV_9p1	CCACCCCGTGTGTACTTACA
FWD_9p2	GGTGGGGCACACATGAATGT	REV_9p2	AATTGGTACGAACGCACTCG
FWD_10p1	TTTATCAATGAAATAATATA	REV_10p1	CAGAAGGTTTTCCCTCATAG
FWD_10p2	GTCTTCCAAAAGGGAGTATC	REV_10p2	AAATAGTTACTTTATTATAT
FWD_11p1	GGCTAAGTAAATCGTAATTC	REV_11p1	CTATACCTCTCGACTGACAC
FWD_11p2	GATATGGAGAGCTGACTGTG	REV_11p2	CCGATTCATTTAGCATTAAG
FWD_12p1	TCCATGTCAGAGAACCCATG	REV_12p1	ATCAAAAGAGAGAGCCGTAG
FWD_12p2	TAGTTTTCTCTCTCGGCATC	REV_12p2	AGGTACAGTCTCTTGGGTAC
FWD_13p1	TAACTCTTCCAGCCACAATC	REV_13p1	CCCGGCCGATCGGGTGTTCG
FWD_13p2	GGGCCGGCTAGCCCACAAGC	REV_13p2	ATTGAGAAGGTCGGTGTTAG
FWD_14p1	GGGGCTGTACTCATGAATGG	REV_14p1	GAGACCGGTTTATCGAGGAC
FWD_14p2	CTCTGGCCAAATAGCTCCTG	REV_14p2	CCCCGACATGAGTACTTACC

Supplementary Table 1. Chiral PCR experimental design. (Upper) Human genome sequence (*fwd* template) and corresponding mirrored chiral DNA sequence (*rev* template) with targeted amplicons in the *fwd* template (colored regions), and their corresponding sites within *rev* template (matched colors) indicated. (Lower) *fwd* PCR primers targeting *fwd* template sequence (left) and mirrored *rev* PCR primers targeting *rev* template sequence (right) indicated.

			Alignment to combined index (hg38/hg38-rev)		
			Human Genome (hg38)	Chiral Genome (hg38-rev)	
LIBRARY	METHOD	READS	Alignments	Alignments	Bacterial origin (BLAST)
Human (*fwd*)	Simulated	731733748	731733671 (99.99%)	77 (1.05 x 10^{-5}%)	0
Chiral (*rev*)	Simulated	731729281	133 (1.8 x 10^{-5}%)	731729148 (99.99%)	0
NA12878 rep1	WGS	35868478	35712395 (99.56%)	66224 (0.18%)	66192 (99.95%)
NA12878 rep2	WGS	37546337	37472078 (99.80%)	145 (3.64 x 10^{-4}%)	101 (69.66%)
NA12878 rep3	WGS	39795112	39706505 (99.78%)	138 (3.47 x 10^{-4}%)	116 (84.06%)
NA12878 rep4	WGS	48854293	48628871 (99.54%)	95288 (0.20%)	95254 (99.96%)

Supplementary Table 2. Alignment statistics for human and chiral DNA libraries. Alignment counts are shown for simulated and experimental libraries (125bp, paired-reads) derived from the human genome, and the chiral genome. For reads in the human WGS libraries that were erroneously cross-aligned to *hg38-rev*, the number that were found to be of bacterial origin (by BLAST search) is also shown.

	Paired-end reads				Single-end reads			
	Chiral Genome (hg38-rev)		Human Genome (hg38)		Chiral Genome (hg38-rev)		Human Genome (hg38)	
READ LENGTH	Total (%)	MapQ > 10 (%)	Total (%)	MapQ > 10 (%)	Total (%)	MapQ > 10 (%)	Total (%)	MapQ > 10 (%)
125	100.0000	92.0509	0.0000	0.0000	99.9999	88.8996	0.0001	0.0000
100	100.0000	91.5546	0.0000	0.0000	99.9995	88.6305	0.0005	0.0000
90	100.0000	91.3160	0.0000	0.0000	99.9991	88.1458	0.0009	0.0000
80	100.0000	91.0191	0.0000	0.0000	99.9983	87.3291	0.0018	0.0000
70	100.0000	90.6597	0.0000	0.0000	99.9964	86.4147	0.0038	0.0000
60	100.0000	90.2260	0.0000	0.0000	99.9927	84.8695	0.0072	0.0000
50	99.9998	89.6338	0.0002	0.0000	99.9801	82.4952	0.0159	0.0001
40	99.9993	88.8516	0.0006	0.0000	99.9224	80.2156	0.0392	0.0002
30	99.9853	87.3789	0.0033	0.0000	96.8352	72.0403	0.1101	0.0001

Supplementary Table 3. Alignment specificity for human and chiral DNA libraries at different read lengths. 30x whole-genome libraries were simulated from the chiral genome (*hg38-rev*) and aligned to combined *hg38/hg38-rev* genome reference. Paired-end and single-end sequencing was simulated across a range of read-lengths (30-125bp). Reported are the total and unique alignments (MapQ>10) to either *hg38* or *hg38-rev*.

Marker	Primer 1 (5' to 3')	Primer 2 (5' to 3')	Reference
BAT-25	TCGCCTCCAAGAATGTAAGT	TCTGCATTTTAACTATGGCTC	Papadopoules *et al.*, 1995.
BAT-25 chiral	AGACGTAAAATTGATACCGAG	AGCGGAGGTTCTTACATTCA	
BAT-26	TGACTACTTTTGACTTCAGCC	AACCATTCAACATTTTTAACCC	Papadopoules *et al.*, 1995.
BAT-26 chiral	TTGGTAAGTTGTAAAAATTGGG	ACTGATGAAAACTGAAGTCGG	
D5S346	ACTCACTCTAGTGATAAATCG	AGCAGATAAGACAGTATTACTAGTT	Spirio *et al.*, 1991.
D5S346 chiral	TGAGTGAGATCACTATTTAGC	TCGTCTATTCTGTCATAATGATCAA	
D2S123	AAACAGGATGCCTGCCTTTA	GGACTTTCCACCTATGGGAC	Weissenbach *et al.*, 1992.
D2S123 chiral	CCTGAAAGGTGGATACCCTG	TTTGTCCTACGGACGGAAAT	
D17S250	GGAAGAATCAAATAGACAAT	GCTGGCCATATATATATTTAA	Weber *et al.*, 1990.
D17S250 chiral	CCTTCTTAGTTTATCTGTTA	CGACCGGTATATATATAAATT	

Supplementary Table 4. Primer sequences for chiral microsatellite profiling. Diagnostic human and chiral primer-pairs used to profile human microsatellite markers and chiral microsatellite standards.

SUPPLEMENTARY REFERENCES

1. Orlov, Y. L. & Potapov, V. N. Complexity: an internet resource for analysis of DNA sequence complexity. *Nucleic Acids Res.* **32,** W628–W633 (2004).

2. Papadopoules *et al.* Mutations of GTBP in genetically unstable cells. *Science* **268,** 1915-1917 (1995)

3. Spirio *et al.* A CA repeat 30-70 kb downstream for the adenomatous polyposis coli (APC) gene. *Nucleic Acids Res*. **19,** 6348 (1991).

4. Weber *et al.* Dinucleotide polymorphisms at the D17S250 and D17S261 loci. *Nucleic Acids Res.* **18,** 4640 (1990).

5. Weissenbach *et al.* A second generation linkage map of the human genome. *Nature* **359,** 794-801 (1992).
