# Supplementary material for: Chiral DNA sequences as commutable controls for clinical genomics
Source: Nat Commun. 2019 Mar 22;10:1342. doi: 10.1038/s41467-019-09272-0 (PMC6430799; doi:10.1038/s41467-019-09272-0)
Supplement: Supplementary file 3 — Reporting Summary [file 41467_2019_9272_MOESM3_ESM.pdf]

nature research

Corresponding author(s): Dr Tim Mercer
Dr Ira Deveson

Reporting Summary

Nature Research wishes to improve the reproducibility of the work that we publish. This form provides structure for consistency and transparency in reporting. For further information on Nature Research policies, see Authors & Referees and the Editorial Policy Checklist.

Statistical parameters

When statistical analyses are reported, confirm that the following items are present in the relevant location (e.g. figure legend, table legend, main text, or Methods section).

n/a	Confirmed	
☐	☒	The exact sample size (*n*) for each experimental group/condition, given as a discrete number and unit of measurement
☐	☒	An indication of whether measurements were taken from distinct samples or whether the same sample was measured repeatedly
☐	☒	The statistical test(s) used AND whether they are one- or two-sided<br>*Only common tests should be described solely by name; describe more complex techniques in the Methods section.*
☒	☐	A description of all covariates tested
☒	☐	A description of any assumptions or corrections, such as tests of normality and adjustment for multiple comparisons
☐	☒	A full description of the statistics including central tendency (e.g. means) or other basic estimates (e.g. regression coefficient) AND variation (e.g. standard deviation) or associated estimates of uncertainty (e.g. confidence intervals)
☒	☐	For null hypothesis testing, the test statistic (e.g. *F*, *t*, *r*) with confidence intervals, effect sizes, degrees of freedom and *P* value noted<br>*Give P values as exact values whenever suitable.*
☒	☐	For Bayesian analysis, information on the choice of priors and Markov chain Monte Carlo settings
☒	☐	For hierarchical and complex designs, identification of the appropriate level for tests and full reporting of outcomes
☐	☒	Estimates of effect sizes (e.g. Cohen's *d*, Pearson's *r*), indicating how they were calculated
☐	☒	Clearly defined error bars<br>*State explicitly what error bars represent (e.g. SD, SE, CI)*

Our web collection on statistics for biologists may be useful.

Software and code

Policy information about availability of computer code

Data collection	None
Data analysis	TrimGalore (v0.4.1), BWA (v0.7.16), Picard (v2.14), GATK (v4.0), GATK (v3.8), Strelka2 (v2.8.4), Ensembl Variant Effect Predictor tool (https://asia.ensembl.org/Tools/VEP), ART (v2.5.8), minimap2 (v2.7), ONT Albacore Sequencing Pipeline Software (version 1.2.6), bamtools piledriver (v2.2.2)

For manuscripts utilizing custom algorithms or software that are central to the research but not yet described in published literature, software must be made available to editors/reviewers upon request. We strongly encourage code deposition in a community repository (e.g. GitHub). See the Nature Research guidelines for submitting code & software for further information.

April 2018

Data

Policy information about availability of data

All manuscripts must include a data availability statement. This statement should provide the following information, where applicable:

- Accession codes, unique identifiers, or web links for publicly available datasets
- A list of figures that have associated raw data
- A description of any restrictions on data availability

All associated data files in including synthetic sequences, variant annotations and sequencing libraries can be accessed at http://www.sequin.xyz/.

Field-specific reporting

Please select the best fit for your research. If you are not sure, read the appropriate sections before making your selection.

☒ Life sciences ☐ Behavioural & social sciences ☐ Ecological, evolutionary & environmental sciences

For a reference copy of the document with all sections, see nature.com/authors/policies/ReportingSummary-flat.pdf

Life sciences study design

All studies must disclose on these points even when the disclosure is negative.

Sample size	For most experiments, three technical replicates were performed, to enable comparison of technical variation between assays.
Data exclusions	No data was excluded without description in the Methods section
Replication	There are not reported findings that failed to reproduced upon experimental replication
Randomization	Not applicable to our experimental design
Blinding	Not applicable to our experimental design

Reporting for specific materials, systems and methods

Materials & experimental systems

n/a	Involved in the study	
☐	☒	Unique biological materials
☒	☐	Antibodies
☒	☐	Eukaryotic cell lines
☒	☐	Palaeontology
☒	☐	Animals and other organisms
☒	☐	Human research participants

Methods

n/a	Involved in the study	
☒	☐	ChIP-seq
☒	☐	Flow cytometry
☒	☐	MRI-based neuroimaging

Unique biological materials

Policy information about availability of materials

Obtaining unique materials	All synthetic sequences and reference materials used in the manuscript are available from commercial vendors or by request from the authors. Sequin spike-in controls are available free for non-profit research.
