# Supplementary material for: Chiral DNA sequences as commutable controls for clinical genomics
Source: Nat Commun. 2019 Mar 22;10:1342. doi: 10.1038/s41467-019-09272-0 (PMC6430799; doi:10.1038/s41467-019-09272-0)
Supplement: Supplementary file 4 — Description of Additional Supplementary Files [file 41467_2019_9272_MOESM4_ESM.pdf]

Description of Additional Supplementary Files

Supplementary Data 1. Somatic variant candidates detected in patient samples and chiral standards.

Identity, confidence metrics and orthogonal validation status for filtered somatic variant candidates in patient tumor samples and internal chiral standards. True-positive (blue) and false-positive (red) candidates detected in chiral standards are distinguished.

Supplementary Data 2. Functional annotation for false-positive and spurious variant candidates.

Functional annotations generated by the Ensembl Variant Effect Predictor for false-positive somatic variant calls in chiral standards and spurious variant candidates detected in patient tumor samples.
